# Effect of governor vessel moxibustion (GVM) therapy with mild to moderate psoriasis: A randomized clinical trial

**DOI:** 10.1097/MD.0000000000035726

**Published:** 2023-10-27

**Authors:** Dan Sun, Wen-Ya Feng, Jing-Kang Mou, Lu Chen, Yi-Ding Zhao, Xiao-Ning Yan, Wen-Bin Li

**Affiliations:** a Department of Dermatology, Shaanxi Provincial Hospital of Chinese Medicine, Xi’an, China; b First Clinical Medical College, Shaanxi University of Chinese Medicine, Xianyang, China

**Keywords:** DLQI, governor vessel moxibustion, moderate psoriasis, PASI

## Abstract

**Background:**

It was hypothesized that governor vessel moxibustion (GVM) therapy may improve the course of mild to moderate psoriasis (PS) in patients.

**Methods:**

A randomized, controlled clinical trial lasting 40 days was conducted at the Shaanxi Provincial Hospital of Chinese Medicine. Investigators were blinded to patient groupings. Individuals with mild to moderate PS ranging in age from 18 to 70 years were enrolled. GVM therapy was administered one every 10 days for 40 days with 1.5 hours on the governor meridian in the GVM therapy group. The PS area and severity index (PASI) and dermatological life quality index (DLQI) scores were monitored before and after treatment.

**Results:**

There was a significant reduction in the mean PASI score in the GVM therapy group of 0.76 points (2.37 [2.61]; SE, 0.39) after 40 days of treatment compared with the control group (3.12 [2.12], SE, 0.32) (*P* < .01). There were also significantly greater changes in the DLQI scores of the GVM therapy group (4.23 [2.25]; SE, 0.34) compared with those in the control group (8.91 [3.85]; SE, 0.59) (*P* < .001).

**Conclusion:**

GVM therapy effectively reduced both PASI and DLQI scores in patients with mild to moderate PS.

## 1. Introduction

Psoriasis (PS) is a recurring and refractory skin illness that can affect the scalp, face, nails, genitals, limbs, and even the entire body, causing scaly erythema, thin film phenomena, and spotty hemorrhaging. PS impacts the physical and mental health of patients, especially when it is associated with cardiovascular disease, obesity, mental and emotional issues, or other diseases.^[1–3]^ Both the occurrence and development of PS are related to heredity, immunity, infection, metabolism, and other factors.^[4]^ Immune dysfunction and interactions in the inflammatory cytokine network contribute significantly to PS pathogenesis and thus form an essential theoretical foundation for the prevention and treatment of the disorder. Targeted drugs such as monoclonal antibodies against interleukin 17 (IL-17) and tumor necrosis factor-alpha (TNF-α, as well as traditional drugs such as retinoic acid, glucocorticoids, calcineurin inhibitors are often not acceptable to PS patients due to the risk of side effects. Although many of these drugs are initially effective.^[5]^ Methotrexate, ciclosporin, vitamin D3 derivatives, retinoids, and glucocorticoids can cause recurrence, irritation, and rebound after withdrawal. They also have anti-inflammatory or immunosuppressive effects on keratinocytes. Targeted biologics are complicated by adverse effects, drug resistance, and price. It is thus essential to find a safe, effective, and affordable PS treatment.

The idea of preventing diseases is gaining ground in the medical world, including the use of advanced traditional Chinese medicine (TCM) for the prevention and treatment of disease. Summer cure winter disease is used to treatment in summer canicular days (dog days) of diseases that show onset or exacerbation during winter.^[6]^ PS patients widely accept governor vessel moxibustion (GVM) therapy, 1 treatment of summer cure winter disease, for its remarkable curative effect, simple operation, and noninvasive and negligible toxic side effects. GVM therapy can be anti-inflammatory by down-regulating the erythrocyte sedimentation rate and C-reactive protein or increasing the total number of CD3 and CD4.^[7]^ Therefore, moxibustion is a promising and valuable approach for PS treatment.

This study aimed to evaluate the effect of GVM therapy on PS using the dermatology life quality index (DLQI), the PS area and severity index (PASI), and the degree of symptom recurrence in patients with mild to moderate PS. The clinical results of GVM treatment in PS patients were evaluated while ELISAs were used to assess the effects on inflammatory factor levels in peripheral blood of PS patients.

## 2. Methods

### 2.1. Study design

The design of the study was a 2-arm blank controlled trial with baseline variables (ratio, 1:1). The study was approved by the Shaanxi Provincial Hospital of Chinese Medicine Ethics Committee for Clinical Research (Ethics Approval Number: (2019) Ethics Review No. 26) and there were no modifications to the protocol after the initiation of the study.

### 2.2. Participants in the study

The inclusion criteria were patients with mild to moderate PS (Supplementary File 3, http://links.lww.com/MD/K459) aged between 18 and 70 years,^[8]^ with PASI scores ≤10 and DLQI scores ≤10. Criteria for recurrence were referred to the advisory group report.^[9]^ All participants provided written informed consent. The participants were required to use topical calcipotriol ointment twice daily. The exclusion criteria were the use of systemic immunosuppressive medications, acitretin, methotrexate, cyclosporin, UV phototherapy, allergy to ginger, moxa, or calcipotriol preparations, concurrent diagnosis of severe medical conditions, mental illness, or pregnancy. A detailed list of the exclusion criteria is provided in Supplementary File 1, http://links.lww.com/MD/K457.

### 2.3. Participant recruitment and randomization

Five physicians (Wen-Bin Li and 4 other dermatologists) with competence in dermatology recruited patients aged 18 to 70 years between 2019 and 2021 from a single outpatient dermatology clinic, where their PASI and Yang deficiency syndrome scores (YDSS) were examined (Supplementary File 2, http://links.lww.com/MD/K458). Subjects underwent the DLQI assessment and peripheral blood collection after providing informed consent. The patients were then randomly allocated to either the control arm or one of the 2 trial arms (GVM therapy) using a computerized randomization list.

### 2.4. Interventions

**Figure 1.**
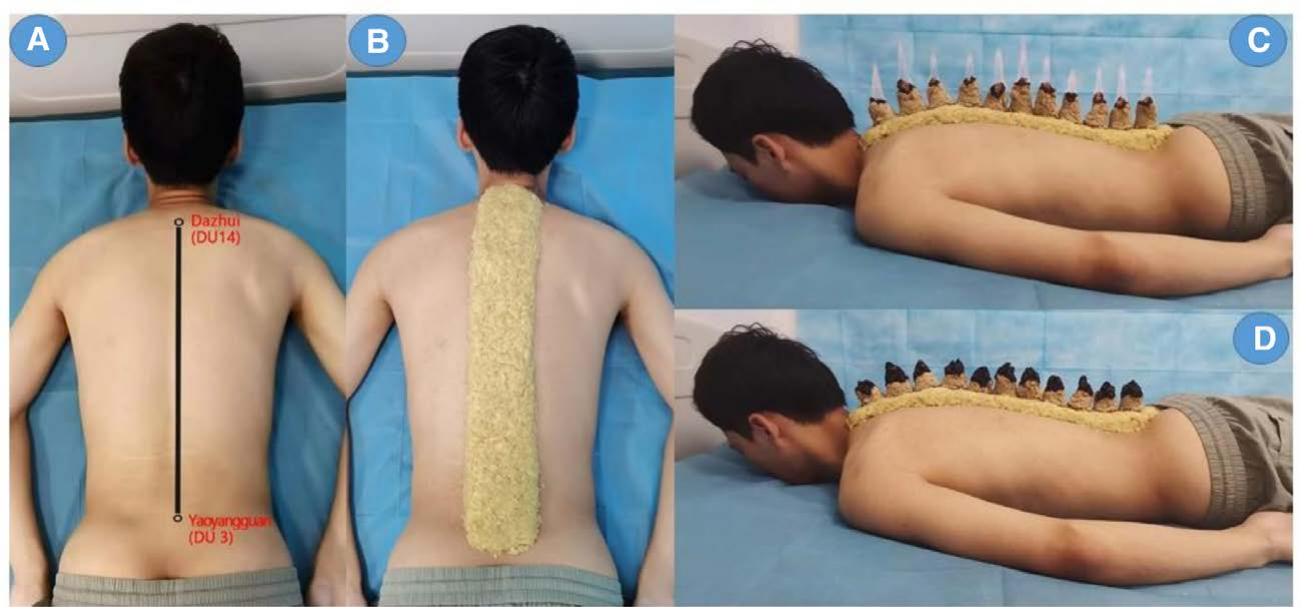
Governor vessel moxibustion (GVM) treatment: (A) acupoints selection; (B) spread ginger at the selected acupoints of the Governor vessel; (C) light the cone of moxa-wool; (D) waiting for the moxa-wool to burn out and remove the ashes.

During the 40-day study period, all patients were treated with a topical Carpotriol formulation following the PS management recommendations. Every 10 days for 4 sessions, participants in the observation group received 1.5 hours of moxibustion, while the control group received no treatment. The GVM treatment was prepared with 1500 g of ginger and 150 g of moxa. Each column of moxibustion was conducted for 30 minutes, with continuous moxibustion for 3 columns at the end of the treatment, with moxibustion treatment during the dog days of summer (July and August). The procedure is shown in Figure 1.

From Dazhui to Yaoyangguan, the treatment area was covered in turmeric and arranged in the shape of a snake (7 × 4 cm) (Fig. 1). The moxa was then formed into a cone (5 × 3 cm), positioned along the center of the ginger paste, and burned it until it was extinguished. Two changes of moxa cones were used. Finally, gauze was used to clean the treatment region. Each treatment lasted around 1.5 hours. Overall, patients received 4 treatments every 10 days for the duration of the trial.

### 2.5. Outcome measures

Change in the PASI and DLQI scores at baseline, 1 month, 40 days after treatment, and at 3 and 6 months of follow-up, as well as the Yang deficiency syndrome score at baseline and 40 days after treatment, were compared between the study arms to determine the treatment effectiveness. Laboratory results served as secondary outcomes. Interferon-gamma (IFN-γ), TNF-α, IL-4, IL-10, and IL-17 levels were measured in peripheral blood samples at baseline and after 40 days of treatment.

### 2.6. Statistical analysis

**Figure 2.**
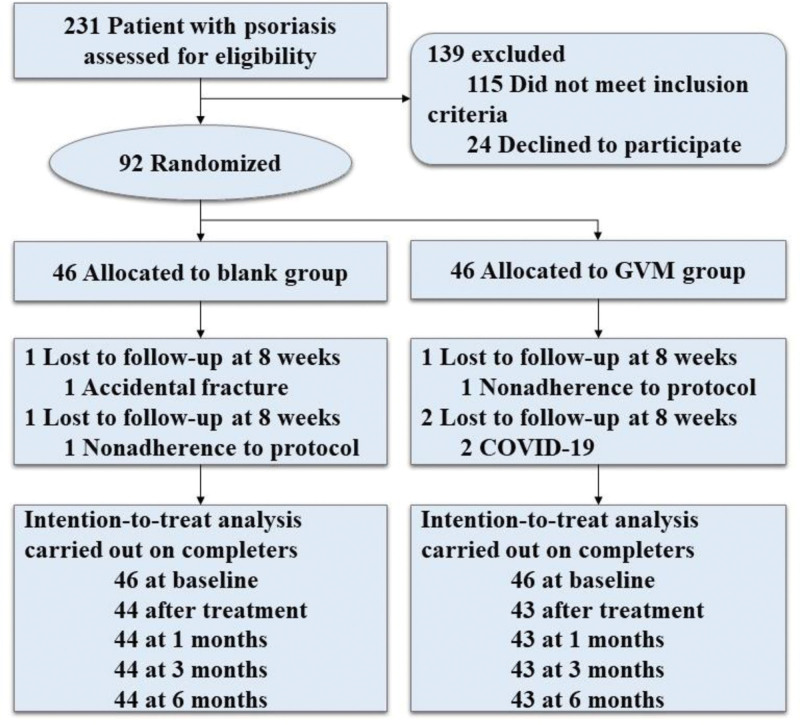
CONSORT flow diagram.

We adhered to the CONSORT guidelines for randomized clinical trials (Fig. 2). The normality of data distribution and homogeneity of variance were assessed. Clinical indicators are reported as mean ± SD. Count data were analyzed by Chi-squared tests. A multivariate ANOVA was used to analyze data from repeated measurements between groups, and groups were compared using *t* tests. *P* values < .05 were considered statistically significant.

### 2.7. Ethical considerations

Ethics was considered by this research team, and this study was initiated after being approved by the Ethics Committee of Shaanxi Provincial Hospital of TCM. Privacy is guaranteed for all patients.

## 3. Results

### 3.1. Clinical indicators

Ninety-two participants with a diagnosis of PS were recruited from July 2019 to August 2021. There were no significant differences between the 2 groups at the baseline (Table 1). The 92 PS patients included 47 males(51.09%) and 45 females(48.91%), ranging in age from 18 to 64 years old (39.522 ± 12.13) with a disease duration ranged from 1 to 35 years (12.65 ± 8.85). One participant left the trial due to an accidental fracture and another was excluded due to non-adherence. In the control group, 1 participant left due to non-adherence, and 2 others due to COVID-19. Table 1 summarizes the severity of disease parameters, including the PASI, DLQI, YDSS, and the laboratory results (IFN-γ, TNF-α, IL-4, IL-10, and IL-17). Before GVM treatment, PS patients were defined as “before,” and after GVM treatment, they were characterized as “after.” There were no significant differences between the GVM and control groups at baseline were statistically negligible (*P* > .05).

**Table 1:** Baseline clinical characteristics and governor vessel moxibustion (GVM) treatment effects of study participants in the blank and GVM groups.

Parameter	GVM group		Blank group		*P*			
	Before (n = 46)	After (n = 44)	Before (n = 46)	Afte r(n = 43)				
Male sex, No.(%)	23 (50.00)	22 (50.00)	24 (52.17)	22 (51.16)	1.000^e^			
Age, mean ± SD, yr	40.89 ± 12.60	NA	38.15 ± 11.62	NA	.281^b^			
Course, mean ± SD, yr	14.15 ± 10.35	NA	11.15 ± 6.81	NA	.104^b^			
PASI, mean ± SD, s	5.65 ± 3.81	2.22 ± 2.42	6.11 ± 2.77	3.22 ± 2.11	.509^b^	.044^a^	<.001^c^	<.001^c^
DLQI, mean ± SD, s	11.57 ± 3.90	4.23 ± 2.25	13.07 ± 4.06	8.91 ± 3.85	.074^b^	<.001^a^	<.001^c^	<.001^c^
YDSS, mean ± SD, s	55.52 ± 15.49	25.96 ± 10.03	58.61 ± 14.71	59.14 ± 14.55	.330^b^	<.001^a^	<.001^c^	.365^d^
Mean (SD), pg/mL						<.001^a^	<.001^c^	<.001^c^
IFN-γ	310.79 ± 45.93	215.88 ± 27.07	295.46 ± 37.03	276.00 ± 37.09	.091^b^	<.001^a^	<.001^c^	<.001^c^
TNF-α	391.79 ± 64.43	273.35 ± 35.24	381.82 ± 58.38	333.65 ± 57.50	.452^b^	<.001^a^	<.001^c^	<.001^c^
IL-4	511.75 ± 74.55	909.46 ± 161.27	532.76 ± 73.74	644.07 ± 75.87	.190^b^	<.001^a^	<.001^c^	<.001^c^
IL-10	152.09 ± 13.09	188.31 ± 21.67	147.59 ± 11.89	155.87 ± 12.22	.097^b^	<.001^a^	<.001^c^	<.001^c^
IL-17	405.15 ± 62.00	278.93 ± 33.45	399.03 ± 64.60	375.77 ± 61.69	.653^b^	<.001^a^	<.001^c^	<.001^c^

a,b Independent samples *t* test, ^a^comparison of GVM group and blank group after treatment, ^b^comparison of GVM group and blank group at baseline; ^c,d^Paired-samples *t* test, ^c^comparison of GVM group before and after treatment, ^d^comparison of blank group before and after treatment; ^e^Pearson chi-square test, χ^2^ = 0.043, *P* = 1.000.

DLQI = dermatology life quality index, GVM = governor vessel moxibustion, IFN-γ = interferon gamma, IL = interleukin, NA = not applicable, PASI = psoriasis area and severity index, s = scores, SD = standard deviation, TNF-α = tumor necrosis factor alpha, YDSS = Yang deficiency syndrome score, yr = years.

### 3.2. Influences on disease severity

**Figure 3.**
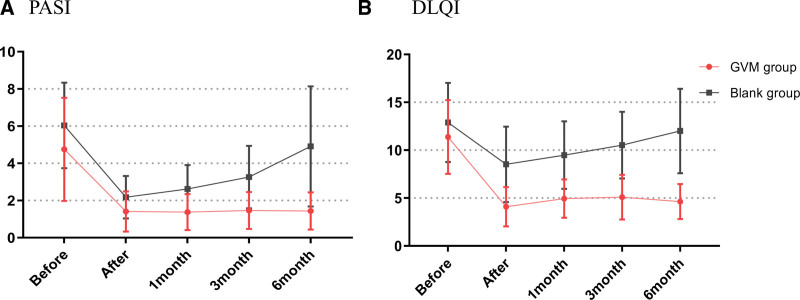
Changes in psoriasis area and severity index (PASI) and dermatological life quality index (DLQI).

**Figure 4.**
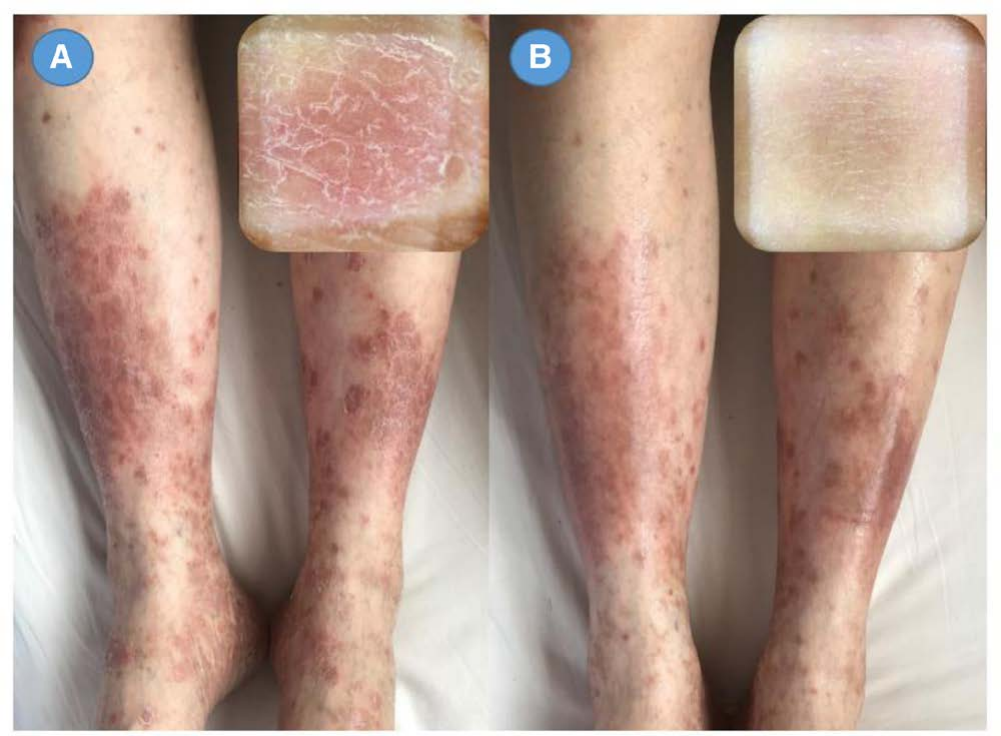
Photos of the psoriatic patients before and after treatment: (A) erythematous scales with infiltrates prior to treatment; (B) lesions pale red with fine lines after treatment.

In the GVM group, 44 out of 46 patients (96%), and in the control group, 43 out of 46 patients (93%) finished the study. Figure 3 displays the mean reduction and 95% CI (confidence interval) for the PASI and DLQI over different weeks. Figure 4 shows that the lesions of PS had improved significantly after treatment. The mean PASI reduction in the PASI in the GVM therapy group was 0.99 points (2.23 [2.42]; SE, 0.37) less after 40 days of treatment compared with the control group (3.22 [2.11], SE, 0.32) (*P < *.001). There was a greater change in the DLQI in the GVM therapy group (4.23 [2.25]; SE, 0.34) compared with the control**s** (8.91 [3.85]; SE, 0.59) (*P < *.001), while the YDSS were significantly lower in the GVM therapy group (25.96 [10.03]; SE, 1.51) than in the control group (59.14 [14.55]; SE, 2.22) (*P < *.001) (Table 1).

### 3.3. Effect on recurrence

When compared to the control group (15 of 27 patients [55.56%]), there was a significant decrease in recurrence in the GVM therapy group (1 of 32 patients [3.1%]) (χ^2^ = 20.37, *P* < .001) (Table 2). GVM treatment could thus prevent the disease from returning compared to the control group and enhance the patient quality of life (Fig. 3).

**Table 2:** PS Recurrence of study participants in the blank and GVM groups.

Parameter	GVM group (n = 32)	Blank group (n = 27)
Recurrence, No. (%)	1 (3.1)	15 (55.6)
No recurrence, No. (%)	31 (96.9)	12 (44.4)

Pearson chi-square test, χ^2^ = 20.368, *P* < .001.

GVM = governor vessel moxibustion.

### 3.4. Comparison of blood marker levels

Table 1 lists the patients’ baseline blood marker levels for the GVM therapy and control groups. Substantial changes in the levels of IFN-γ, TNF-α, IL-4, IL-10, and IL-17 blood levels were seen between the 2 groups during the 40-day intervention period.

### 3.5. Negative effects

There were no treatment-related adverse effects.

## 4. Discussion

The effectiveness of moxibustion in preventing and treating PS has been the subject of several earlier studies.^[10,11]^ Therefore, the available data point to moxibustion as a potential treatment to increase the rate of patient recovery from for mild and moderate PS. However, there are no reliable experimental data on the efficacy and safety of moxibustion in clinical settings. Improving the quality of life of PS patients and integrating disease prevention and therapy is crucial. The principle of TCM is that evil qi cannot attack the body echoes the role of immunological regulation in maintaining human health. All diseases are caused by insufficient Yang Qi to protect the body.^[12]^ Yang deficiency causes severe winter PS and mild summer PS. Excessive use of TCM for clearing heat and decreasing fire, and incorrect use of antibiotics, glucocorticoids, and immunosuppressants will affect human Yang Qi and lead to an increase in Yang deficiency syndrome, even joint pain, and deformity in PS. Therefore, supplementing Yang Qi to prevent and control disease transmission and change is an essential principle of TCM treatment of patients with PS with positive syndromes.

Governor vessel moxibustion, also known as long snake moxibustion, is a traditional non-drug therapy in TCM. It is the primary representative therapy of warm Yang to remove pathogenic factors^.[13]^ As the sea of the Yang vessel, the governor vessel can be unified supervision of Yang Qi the whole body and is the key to the coordination of Yin and Yang.^[14]^ The canicular days are the time when Yang Qi reaches its peak according to TCM theory. The canicular days belong to the golden lungs of the 5 elements, responsible for the skin opening and closing.^[15]^ Hence, we implement GVM treatment to strengthen the role of warming Yang and to activate vessel circulation in the canicular days.

GVM therapy has the advantages of warm Yang Qi and smooth veins, and is relatively friendly to PS patients who are aggravated in winter or aggravated by cold. On the contrary, it is not suitable for distinguishing as heat syndrome PS people and the PS in special periods, resulting in the risk of miscarriage in pregnant women or increased menstrual flow in menstrual women.

GVM therapy for 40 days was found to reduce the PASI, DLQI, and YDSS significantly more than those of the control group. GVM treatment reduced the recurrence of PS, increased the quality of life, and modified inflammatory variables such as IL-17, IL-10, IL-4, TNF-α, and IFN-γ. GVM treatment thus improves patients’ quality of life by reducing psoriatic lesions and inflammation.

## 5. Conclusion

GVM therapy effectively reduced the PASI and DLQI scores, reduced PS recurrence, and regulated the levels of IL-17, IL-10, IL-4, TNF-α, and IFN-γ in the peripheral blood of patients with mild to moderate PS.

### 5.1. Limitations and future research recommendations

The curative effect of GVM in treating severe PS with non-yang deficiency syndrome was not addressed as this study did not evaluate the curative effect of GVM in treating severe PS and other syndromes. The next step will be for the research team to further investigate the efficacy of GVM treatment in PS in addition to the canicular days (dog days) cycle.

## Author contributions

**Conceptualization:** Lu Chen.

**Data curation:** Wen-Ya Feng.

**Methodology:** Lu Chen.

**Software:** Jing-Kang Mou.

**Validation:** Yi-Ding Zhao, Wen-Bin Li.

**Visualization:** Jing-Kang Mou, Xiao-Ning YAN, Wen-Bin Li.

**Writing – original draft:** Dan Sun.

**Writing – review & editing:** Dan Sun, Wen-Bin Li.

## Supplementary Material






