# Supplementary material for: Effect of governor vessel moxibustion (GVM) therapy with mild to moderate psoriasis: A randomized clinical trial
Source: Medicine (Baltimore). 2023 Oct 27;102(43):e35726. doi: 10.1097/MD.0000000000035726 (PMC10615393; doi:10.1097/MD.0000000000035726)
Supplement: Supplementary file 2 [file medi-102-e35726-s002.docx]

**Supplemental Digital Content**

**Supplement 1: Study Protocol**

**1. The main target**

To observe the recurrence of psoriasis, PASI score and quality of life scale score of patients with yang-deficiency psoriasis after Governor vessel moxibustion (GVM) therapy with Summer cure winter disease (SCWD).The changes of serum cytokines related to psoriasis were detected before and after treatment.

**2. Research and development content**

(1) A randomized controlled clinical trial was designed;

(2) To observe the efficacy and safety evaluation of GVM therapy in the treatment of Yang deficiency psoriasis;

(3) Follow up patients who were cured and had significant effect in clinical trials to evaluate the long-term efficacy.

**2.1 The research object**

**2.1.1 Source of case**

The patients with psoriasis was from Shaanxi Provincial Hospital of Chinese Medicine.

**2.1.2 Western diagnostic criteria**

Refer to the French guidelines on the use of systemic treatments for moderate-to-severe psoriasis in adults.

(1) Occured in young and middle-aged people, or caused by upper respiratory tract infection.

(2) Occured on the scalp, trunk, and limbs, and distributed or confined to a single location.

(3) A miliary to mung bean size red papule, macules, or plaques were typical lesions, with well-defined margins and marked infiltration. Clinical features included white scaling, thin film and punctate bleeding.

(4) Bundle in the scalp, or accompanied by finger (toe) armour, mucous membrane damage.

(5) Three stages in clinical stages: progressive stage, static stage and regression stage.

(6) Chronic course of disease, or even lifelong delay. Winter recrudescence or aggravation, spring and summer to reduce or disappear more common.

(7) Munro microabscesses in or below the cuticle, thinning or disappearance of the granular layer, hypertrophy of the spinous layer, lengthening of the epidermal crest, distortion and expansion of blood vessels in the dermal papilla, and mild thickening. The epidermis above the nipple was thinned, and the upper dermis was mildly to moderately inflammatory with pathologic infiltration.

**2.1.3 Yang-deficiency syndrome standard of TCM**

Refer to the expression of Yang deficiency syndrome in TCM Clinical Diagnosis: pale, fear of cold, light tastes, self-sweating, cold fear of limbs, fatigue, lethargy, loose stools, long and clear urine, pale menstruation in female, pale tongue, teeth marks on tongue, fat tongue, moist tongue, moistened tongue coating, heavy pulse.

**2.1.4 Included in the standard**

(1) Meet the diagnostic criteria of psoriasis.

(2) No less than 18 years old and no more than 70 years old, male or female.

(3) No corticosteroids, immunosuppressants, biological agents and other related drugs were used in the last 1 month;

(4) The subject or guardian had signed the informed consent;

(5) Before treatment, routine blood, urine and feces tests, liver and kidney function tests were within the normal range.

**2.1.5 Exclusion criteria**

(1) Had received systemic therapy (actretin, MTX, cyclosporin, UV phototherapy) within 4 weeks before the trial.

(2) Pregnant or lactating women;

(3) Allergy to any ingredient in the test drug;

(4) Patient with serious primary diseases of cardiovascular, cerebrovascular, liver, kidney and hematopoietic system and mental illness patient.

(5) Patient refused to sign the informed consent, or estimated poor compliance, poor possibility of follow-up.

**2.2 Methods**

**2.2.1 Study Design and Population**

The study design was a double-blind, 2-arm blank controlled trial by baseline variables (ratio, 1:1). The study received approval from the Ethics Committee for Clinical Research of Shaanxi Provincial Hospital of Chinese Medicine. There was no change to the trial protocol after it commenced.

Patients aged 18 to 70 years were recruited between 2019 and 2021 from a single outpatient dermatological clinic where 5 dermatologists (Wen-bin LI and 2 others) with expertise in dermatology evaluated their PASI and Yang deficiency syndrome score(YDSS) (Supplement 2: Yang deficiency syndrome score). After informed consent was obtained from their legal representatives, participants completed the dermatology life quality index (DLQI) and peripheral blood collection. Patients were then assigned to 1 of the 2 trial arms (GVM therapy or control) following a computerized randomization list.

**2.2.2 Interventions**

All patients received treatment during the 40-day study period with topical Carpotriol preparation according to the guidelines for the management of PS. Participants in the observation group received moxibustion for 1.5 hours every 10 days for 4 times in total, and no intervene in control group.

GVM treatment was prepared with 1500g ginger and 150 moxa, each column of moxibustion was carried out for 30 minutes, continuous moxibustion for 3 columns to end the treatment, and the time of moxibustion should be in the dog days of summer. Refer to Figure 1 for the operation process.

The treatment area was covered with turmeric placed in the shape of a snake (7 cm × 4 cm) from Dazhui (DU 14) to Yaoshu (DU 2). Then, knead the moxa into a cone shape as moxa cone (5cm × 3cm), place the moxa cone along the center of the ginger paste, burn until it goes out. The moxa cone was replaced twice. Eventually, the treatment area was wiped with a gauze. The duration of each treatment was approximately 1.5h. The patients were treated every 10 days for 4 times in total course.

**2.3 Obvervational index**

Including general information (age, sex, etc.), factors affecting the outcome (Such as course, comorbidities, treatment, etc.), medication before treatment, safety indicators (such as blood, urine, fecal routine, liver, kidney, etc.).

**2.4 Measurement**

**(1)Main outcome measures**

PASI(Psoriasis area and severity index), DLQI(The Dermatology Life Quality Index，DLQI), postponing the recurrence and IFN-γ, TNF-α, IL-4, IL-10, IL-17 in peripheral blood were analyzed.

**(2)Secondary outcome measure**

The expressions of IFN-γ, TNF-α, IL-4, IL-10 and IL-17 in peripheral blood of patients with psoriasis were detected by ELISA。

All subjects re-visited at the first visit and the 2nd, 4th, 6th and 8th week of treatment. The skin lesion area, erythema, scale and infiltration degree were recorded and PASI score was calculated. Fill in the DLQI scale and calculate the score. Safety inspection was conducted once before and after treatment, and medication compliance was inquired, and combined medication and adverse reactions were recorded.

**2.5 Efficacy evaluation method**

The treatment improvement rate was calculated as follows: treatment improvement rate = (PASI score before treatment - PASI score after treatment)/PASI score before treatment ×100%. Clinical recovery: all skin lesions disappeared, the total treatment rate ≥90%. Significant effect: most of the lesions disappeared, the total treatment rate of 60%≤ was < 90%. Progress: skin lesions partially subsided, 20%≤ total treatment rate < 60%. Ineffective: no obvious regression of skin lesions, the total efficacy rate < 20%.

**2.6 Laboratory index**

**2.6.1 Security testing**

Routine blood, urine and feces, liver and kidney function were detected in the laboratory of our hospital, and the changes of basic indicators before and after treatment were observed.

**2.6.2 Inflammatory factor detection**

The serum levels of IFN-γ, TNF-α, IL-4, IL-10, IL-17 were determined by ELISA at the baseline and after treatment. All testing steps carried out according to the instructions in the kit.

**2.7 Follow-up**

After the treatment, the main measures and the treatment period were analyzed, to heal and the subjects had marked effect for 6 months follow-up, respectively in 1, 3, 6 months follow-up after treatment. The clinical symptoms and quality of life score changes, monitoring the blood routine, urine routine, liver function, renal function, were tested and analyzed. Criteria for recurrence were referred to the advisory group report(Carey W, Glazer S, Gottlieb AB, et al. Relapse, rebound, and psoriasis adverse events: an advisory group report. J Am Acad Dermatol. 2006, 54(4 Suppl 1): S171-81).

**2.8 Statistical methods**

The clinical indicators were expressed as mean ± standard deviation, which were consistent with the data of normal distribution and homogeneity of variance. The counting data was analyzed by Chi-square test, the comparison between groups was conducted by a t-test, the repeated measurements data between groups was analyzed by the repeated measures ANOVA, P<0.05 was considered statistically significant.
