# Supplementary material for: Effect of governor vessel moxibustion (GVM) therapy with mild to moderate psoriasis: A randomized clinical trial
Source: Medicine (Baltimore). 2023 Oct 27;102(43):e35726. doi: 10.1097/MD.0000000000035726 (PMC10615393; doi:10.1097/MD.0000000000035726)
Supplement: Supplementary file 3 [file medi-102-e35726-s003.docx]

**Supplemental Digital Content**

**Supplement 2: Yang deficiency syndrome score**

The purpose of this questionnaire is to have a more comprehensive understanding of your Yang deficiency syndrome and provide reference for future health management and clinical diagnosis and treatment. Please fill in your basic information first, then read each question carefully and tick "√" in the corresponding "□" according to your actual situation or feeling in the last 4 weeks. If you can't answer "yes" to a question, choose the answer closest to your true feelings. If you have any comments, please let our investigators know.The information you fill in will be kept strictly confidential and will not be disclosed in any form. Thank you for your support and cooperation.

Start time:

| Name: Gender: □Male □Female Age:  Native Place:  | |
| --- | --- |
| Profession | □State organs, Party and mass organizations, Persons in charge of enterprises and institutions |
|  | □Professional and technical personnel □Clerical and associated personnel |
|  | □Production personnel in agriculture, forestry, animal husbandry, fishing and water conservancy industries |
|  | □Production and transportation equipment operators and related personnel |
|  | □Business and service personnel □Retiree |
|  | □Soldier □Student □Other |
| Level of education | □Illiteracy and semiliterate □Primary school □Junior high school |
|  | □Senior high school/ Technical secondary school /Technical school  □Undergraduate/Junior College □Master student  □Doctoral candidate |

Please answer the following questions based on your feelings and experiences in the last 4 weeks.

|  | no  (0) | little  (1) | sometimes  (2) | often  (3) | always  (4) |
| --- | --- | --- | --- | --- | --- |
| 1.Do you have cold hands and feet? | □ | □ | □ | □ | □ |
| 2.Do you feel mentally tired? | □ | □ | □ | □ | □ |
| 3.Do you have any weakness? | □ | □ | □ | □ | □ |
| 4.Do you get cold easily and wear more clothes  than others? | □ | □ | □ | □ | □ |
| 5.Do you like to curl up when you sleep? | □ | □ | □ | □ | □ |
| 6.Does your breath from your nose and mouth  feel cold? | □ | □ | □ | □ | □ |
| 7.Do you have asthma? | □ | □ | □ | □ | □ |
| 8.Do you feel tightness in your chest? | □ | □ | □ | □ | □ |
| 9.Do you have mild and persistent chest pain? | □ | □ | □ | □ | □ |
| 10.Do you have any swelling or tightness in the  middle of your upper abdomen? | □ | □ | □ | □ | □ |

Please answer the following questions based on your feelings and experiences in the last 4 weeks.

|  | no  (0) | little  (1) | sometimes  (2) | often  (3) | always  (4) |
| --- | --- | --- | --- | --- | --- |
| 11.Do you have palpitations easily (fast heart rate)? | □ | □ | □ | □ | □ |
| 12.Do you feel your heart beating violently and uneasily? | □ | □ | □ | □ | □ |
| 13.Do you have mild and persistent pain in your midsection? | □ | □ | □ | □ | □ |
| 14.Do you feel comfortable or relieved if you eat (drink) warm food, or feel uncomfortable or afraid of eating (drink) cold food? | □ | □ | □ | □ | □ |
| 15.Do you expectorate a lot and have foams? | □ | □ | □ | □ | □ |
| 16.Do you catch a cold more easily than others? | □ | □ | □ | □ | □ |
| 17.Don't you have a cold and runny nose? | □ | □ | □ | □ | □ |
| 18.Do you often spit clear and thin when you have a cold? | □ | □ | □ | □ | □ |
| 19.Do you sweat easily with little or no activity (self-sweating)? | □ | □ | □ | □ | □ |
| 20.Do you feel distension? | □ | □ | □ | □ | □ |

Please answer the following questions based on your feelings and experiences in the last 4 weeks.

|  | no  (0) | little  (1) | sometimes  (2) | often  (3) | always  (4) |
| --- | --- | --- | --- | --- | --- |
| 21.Have you been eating less? | □ | □ | □ | □ | □ |
| 22.Do you feel distention in the upper abdomen after eating? | □ | □ | □ | □ | □ |
| 23.You feel a lot of saliva, do you have to spit it out often? | □ | □ | □ | □ | □ |
| 24.Do you have any vomit or water? | □ | □ | □ | □ | □ |
| 25.Do you feel cold at the waist? | □ | □ | □ | □ | □ |
| 26.Do you feel cold in your joints? (e.g. knee joint, shoulder joint, chronojoint, etc.) | □ | □ | □ | □ | □ |
| 27.Do you have any mild and persistent pain in your joints? | □ | □ | □ | □ | □ |
| 28.Do you have cold pain in your waist and/or knees? | □ | □ | □ | □ | □ |
| 29.Do you have pain in your back and/or knees? | □ | □ | □ | □ | □ |
| 30.Do you feel cold in your abdomen? | □ | □ | □ | □ | □ |
| 31.Do you feel cold in the lower abdomen just below the umbilicus? | □ | □ | □ | □ | □ |

Please answer the following questions based on your feelings and experiences in the last 4 weeks.

|  | no  (0) | little  (1) | sometimes  (2) | often  (3) | always  (4) |
| --- | --- | --- | --- | --- | --- |
| 32.Do you have loose stools? | □ | □ | □ | □ | □ |
| 33.Do you often have diarrhea? | □ | □ | □ | □ | □ |
| 34.Do you urinate more often at night or more than others? Do your urine color clear (light)? | □ | □ | □ | □ | □ |
| 35.Do you feel that you have a lot of urine when you urinate? | □ | □ | □ | □ | □ |
| 36.Do you have swelling in your body or limbs? | □ | □ | □ | □ | □ |
| 37.Do you have dark circles under your eyes easily? | □ | □ | □ | □ | □ |
| 38.Do you have mild and persistent pain in your abdomen? | □ | □ | □ | □ | □ |
| 39.Do you have diarrhea in the morning? | □ | □ | □ | □ | □ |
| 40.Do you have indigestible food in your bowel movements? | □ | □ | □ | □ | □ |
| 41.Do you have more stools if you eat greasy or cold food | □ | □ | □ | □ | □ |
| 42.Do you have a loss of libido? | □ | □ | □ | □ | □ |
| 43.Do you have normal sex, have no contraception and have not given birth for 2 years? | □ | □ | □ | □ | □ |

Please answer the following questions based on your feelings and experiences in the last 4 weeks.

|  | no  (0) | little  (1) | sometimes  (2) | often  (3) | always  (4) |
| --- | --- | --- | --- | --- | --- |
| 44.Is your white ribbon as thin as water? (Women only) | □ | □ | □ | □ | □ |
| 45.Is your menstruation thin and pale? (Women only) | □ | □ | □ | □ | □ |
| 46.Have you had more than two consecutive menstrual cycles that were more than 7 days later than normal? (Women only) | □ | □ | □ | □ | □ |
| 47.Do you feel cold in your scrotum? (Men only) | □ | □ | □ | □ | □ |
| 48.Is your ejaculation cold? (Men only) | □ | □ | □ | □ | □ |
| 49.Do you have impotence? (Men only) | □ | □ | □ | □ | □ |
| 50.Do you have no ejaculation due to sexual intercourse or dreaming? (Men only) | □ | □ | □ | □ | □ |

How long did it take you to complete this questionnaire (refer to the "start time" to fill in) about ( ) minutes

The following was filled out by the investigator:

Western medicine diagnosis: TCM diagnosis:

Syndrome type diagnosis:
